# Developing a patient and public involvement intervention to enhance recruitment and retention in surgical trials (PIRRIST): study protocol

**DOI:** 10.1186/1745-6215-16-S2-P89

**Published:** 2015-11-16

**Authors:** Joanna Crocker, Sian Rees, Louise Locock, Sophie Petit-Zeman, Alan Chant, Shaun Treweek, Jonathan Cook, Nicola Farrar, Kerry Woolfall, Richard Bulbulia

**Affiliations:** 1 Health Experiences Institute, University of Oxford, Oxford, UK; 2 NIHR Biomedical Research Centre, Oxford, UK; 3 Health Experiences Research Group, Nuffield Department of Primary Care Health Sciences, University of Oxford, Oxford, UK; 4 NIHR Oxford Biomedical Research Centre and Unit, Oxford, UK; 5 Patient Representative, Berkshire, UK; 6 Health Services Research Unit, University of Aberdeen, Aberdeen, UK; 7 Surgical Intervention Trials Unit, University of Oxford, Oxford, UK; 8 MRC ConDuCT-II Hub for Trials Methodology Research, School of Social and Community Medicine, University of Bristol, Bristol, UK; 9 MRC North West Hub for Trials Methodology Research, Institute of Psychology, Health and Society, University of Liverpool, Liverpool, UK; 10 MRC CTSU Hub for Trials Methodology Research, Clinical Trial Service Unit, Nuffield Department of Population Health, University of Oxford, Oxford, UK

## Background and aims

Slow recruitment and poor retention are common challenges to the successful delivery of clinical trials, particularly surgical trials. Patient and public involvement (PPI) in designing and conducting clinical trials has the potential to enhance recruitment and retention but there have been few attempts at rigorous evaluation. The aim of PIRRIST is to develop a PPI intervention that improves recruitment and/or retention in surgical trials.

## Methods

The study comprises four stages, beginning in July 2015: (1) Mapping current PPI practice in UK surgical trials through a survey and analysis of National Research Ethics Service data; (2) Focus groups with key stakeholders (patients or members of the public involved in surgical trials, surgical trial investigators, administrators and PPI co-ordinators) to explore their needs, the challenges associated with PPI and how PPI might support recruitment and retention; (3) A survey of key stakeholders’ views about the possible components of one or more potential PPI interventions; (4) A consensus workshop with a broad, purposive sample of stakeholders to select the PPI intervention that we put forward for future evaluation.

## Outcomes

This study will lead to a robust, evidence-based PPI intervention ready for evaluation. Although tailored to surgical trials, the findings will enhance understanding of whether and how PPI might improve recruitment and retention in clinical trials.

The study is part of the Trial Forge initiative to improve trial efficiency.

